# Medical Extracts

**Published:** 1884-09

**Authors:** 


### Medical Extracts.

### Case of Lipoma of the Arachnoid.

Dr. Braubach records a case of Lipoma of the Arachnoid of the Cervical Cord, and extending to the point of exit of the 4th and 5th dorsal nerves. The cord was completely compressed and displaced to the left side. Below the region of compression there was descending degeneration of the pyramidal columns.

The nature of the tumour is very rare in this situation. Very few such cases have been recorded: one by Obré, one by Athol Johnson, one by Virchow, and a case of myo-lipoma by Gowers. The paraplegia of the lower limbs, their extreme anæsthesia, the bladder weakness, the extreme contraction of both lower extremities, the increase of their tendon reflexes, need no comment. They depend partly on the compression of the cervico-dorsal cord, partly on the descending secondary degeneration of the lateral pyramidal tracts.

Of greater interest is—

1. The fact that, in spite of the extreme compression and degeneration of the affected part of the cervico-dorsal cord, the conduction through it, at any rate of intense impressions of pain, was still retained.

2. The fact that, in spite of the great degeneration of the compressed parts, the roots on the right side that came off from these affected regions shewed only very slight degeneration, the roots on the left side none.

3. The fact that the left upper extremity could be moved as in health, without any paralysis or contraction; whilst the right arm was paralysed and extremely contracted. The latter symptom was, doubtless, due to the great amount of stretching of the right spinal roots, in consequence of the displacement of the cord to the left; and a permanent state of irritation was present throughout, which gave occasion to tonic contraction of the right upper extremity.—*Arch. für Psych. und Newenkrankeiten*, xv. Band., 2 Hept.

### A Case of Tubercle occupying the middle third of the ascending frontal convolution.

Symptoms: Epileptiform convulsions, and concentric paretic phenomena of the right arm, and of the buccal branch of the facial of the same side. Right-sided deviation of the tongue; slight dilatation of the pupil of that side.

Then the convulsions became general, were accompanied or followed by agitation, disturbances of consciousness, delirium, crying or childish laughter. Death from general tuberculosis.—CHVOSTECK, *Jahrbüch für Psych.*, iv. 1.

### Pharmacology of Hamamelis Virginica.

In an article on the properties and therapeutic uses of Hamamelis, M. Ch. Eloy points out that though the drug has long been known, and to some extent used, the active principles of the plant have remained unknown; but recently M. Van der Espt has laid before the Sociëté Royale des Sciences Médicales of Brussels the results of his investigations into the nature of the drug. Hamamelis contains no alkaloid; it owes its properties to its richness in gallic and tannic acids, which are present along with a red colouring matter and an aromatic substance. As an astringent to mucous membranes and blood-vessels, an extract of the bark must be used; it is not known for certain whether the leaves possess as much astringent power.

Under the name of Hazeline, there is extensively used in England and America a distilled water of Hamamelis, of which the aromatic taste and odour are rather agreeable. The medicinal activity of this preparation has been less certainly demonstrated.—*L'Union Médicale*, August 9th.

[The above is not in accordance with the general opinion in this country; see, for example, Martindale's *Extra Pharmacopœia*, under Hamamelis. M. Dujardin-Beaumetz, at the Société de Thérapeutique, in May last, stated that he had never seen any of the physiological effects described by M. Van der Espt even after large doses.—Rep.]

### Rabies in Birds.

M. Paul Gibier communicated, on July 19th, a note to the Sociétié de Biologie upon Rabies as it occurs in birds. His conclusions are:—

Birds are susceptible to Rabies.

Birds do not have Rabies twice, one attack being prophylactic.

A bird with Rabies can transmit the malady to another bird.

Birds can transmit the malady in a fatal form to mammals, while the birds themselves may recover.

Only when a large dose of the virus is administered to a bird does the disease prove fatal.

The virus of Rabies in passing through a succession of birds seems to increase in the intensity of its effect on birds, but diminish in intensity of effect upon dogs.

In fatal cases he finds the brain crowded with the specific micrococcus of Rabies, which he described for the first time to the Society a year ago.—*L'Union Médicale*, August 28th.

### Prevention of Rabies.

The official Commission, appointed some months ago, to control the discoveries of M. Pasteur as to the prevention of Rabies by "vaccination" with attenuated virus, has issued its report upon the first set of experiments.

Forty-two dogs were experimented upon altogether; 23 were rendered insusceptible of Rabies according to Pasteur's method, and 19 were left without protective inoculation. All the dogs received the Rabies virus, either by being bitten by other mad dogs, by intra-venous injection, or by inoculation by trephining.

Of the 23 dogs who had previously received protective vaccination, not one had developed Rabies at the date of the report. Of the 19 unprotected, 3 (out of 6) which had been bitten by a mad dog, 6 (out of 8) after intra-venous injection, and 5 (out of 5) of those inoculated by trephining, developed Rabies; that is, 14 out of the 19.

The Committee intend to continue to make further experiments, especially upon "vaccinated" dogs. The most important point to be determined, however, from the human point of view, is, whether "vaccination" with attenuated virus is capable of protecting the subject from the effects of virus inoculated by the recent bite of a mad dog.—*L'Union Médicale*, August 14th.

### Iodine and Ergotine in Goitre.

M. le Dr. Bauwens, à Alost, sums up the varieties of goitre as follows:—

1. Goitre may be sporadic. The constitution of the individual is not the cause; it is a local malady, without diathetic cause. Such is the goitre due to mechanical causes, effort, accouchement.

2. It may depend on a blood change, as one of the multiple manifestations of the scrofulous diathesis.

3. It is one of the constituent parts of the triad of symptoms of exophthalmic goitre.

4. It is endemic, as in the deep valleys and narrow gorges of mountainous countries. It is generally allied to other degenerations, and forms part of the symptoms of Cretinism.

5. It exists everywhere epidemically.

After an inquiry into the action of iodine in the different varieties of goitre, he gives the following *resumé* :—

Iodine cures in the endemic, non-degenerated goitre; one may sometimes employ it with success in the soft recent goitres of the scrofulous; it is useless in all degenerated goitres, in the epidemic and in the cystic forms; it is injurious in the sanguineous and exophthalmic varieties.

The author finds that the parenchymatous injection of the tincture of iodine is most useful in cystic goitre, the action being analagous to the similar use of iodine in synovial cysts, mucous bursæ, serous cysts, &c.

The author then inquires into the action of ergotine, and he finds that the general circulation is calmed, the blood supply of the thyroid arteries diminishes considerably, and so gives a first cause of the regression of the vascular tumour. He points out that the thyroid body is a kind of diverticulum to the right heart, and that the more irregular the circulation and the greater the amount of venous stasis, the more also does the gland swell. Ergotine, in regulating the cardiac contractions, restores the equilibrium between the right and the left sides of the heart, and so with the diminution of venous stasis we have a second cause of the regression of the tumour. Such is the general sedative action of the drug; but from parenchymatous injection it is the local action which predominates; and where (the author asks) can ergot better exercise its constricting, contractile action on the smooth muscular fibres which abound in the small blood-vessels than in the thyroid gland, where the fibrous capsule serves more perfectly to localise its effects?

The paper concludes with the following table, giving the indications and contra-indications for the parenchymatous injection of the Tincture of Iodine and of the Ergotine d'Yvon:—

INDICATIONS.

| *Tincture of Iodine.* | *Ergotine d'Yvon.* |
|---|---|
| *a.* Cystic goitre: cure. | Idem. |
| *b.* Soft, diffuse, recent goitre: cure sometimes. | Success is certain. |
| *c.* Vascular sanguineous goitre: it is dangerous and useless. | Success is certain. |
| *d.* Exophthalmic goitre: dangerous. | Its physiological action guarantees success. |
| *e.* Degenerated goitre: injurious. | It has cured, even in case of ossified goitre. |
| *f.* Fibrous goitre: useless. | It may succeed. |
| *g.* Retro-sternal goitre: dangerous. | It should be tried, and will succeed if the tumour be parenchymatous. |
| *h.* Cancerous goitre: useless. | Useless. |

—*Bulletin de l'Académie Royale de Médecine de Belgique*, 1884, Tome xviii., No. 2.

### On Hazeline in Menorrhagia.

"Of the many ailments of women that fall under the notice of practitioners in this colony, menorrhagia may be considered to be a very frequent one. In its many forms it would be perhaps difficult to mention any process that has a surer influence in producing in the first place, and afterwards in maintaining, that condition of languor and debility which is so frequently met with, than this regular draining of blood. I wish to confine my remarks to that form of menorrhagia which begins gradually by an increase in the menstrual flow, creeping on month by month, a slight increase each time, most frequently in the time it lasts, rather than in the quantity lost, which, when continuing unheeded, at last sooner or later —no doubt by congestive mucous swelling—establishes a condition of obstruction, and then pain is felt. As a rule, I have found that it is this pain for which relief is sought; the condition which has established it is not considered anything but natural.

"The main object of my letter is to mention how valuable a remedy the American Witch Hazel, Hamamelis Virginica or Hazeline, is in this disease. It possesses the advantage of acting quickly, with no unpleasant effects, and it is not at all unpleasant to take, sugared water disguising the taste, somewhat resembling benzoline, that it has.

"In doses of half a tea-spoonful, twice or three times a day, its effect has in my experience been most effectual.

"It acts so quickly that it is not necessary to anticipate the flow, but when menstruation, after it has lasted the ordinary time, is not closing naturally, Hazeline, given as above, will effectually restrain it; and after hæmorrhage has ceased there is no advantage in continuing it. While it is taken, some patients have mentioned to me that they have a pleasant sense of exhilaration, of being strung up, and have lost that wearying sense of languor felt at these times.

"Another good result Hazeline produces is, that when there is Dysmenorrhœa, it, in a very quick and marked way, relieves the pain. I have in my recollection the case of a young lady who suffered severely, so much as to necessitate her keeping in bed, who was once so bad as to require a hypodermic injection of morphia. Since she has taken Hazeline menstruation has been painless, and not excessive as formerly.

"It would be interesting to know if the experience of others agrees with mine on the value of Hazeline as an astringent."—HENRY M. CHUTE, *South African Medical Journal*, February 15th, 1884.

### On Fat Embolism.

Dr. Park thus summarises an elaborate paper on "Fat Embolism," in the *New York Medical Journal* of August 16th, 1884:—

1. Fat embolism in varying degrees of severity is not an uncommon complication of surgical accidents and operations.

2. It may be so mild as to be lost sight of in the general condition of shock, or, perhaps, more properly speaking, it is one factor of a condition of prolonged shock.

3. Our knowledge of the subject will be greatly increased when we appreciate the possibilities of its occurrence and observe our cases more closely, watching for the appearance of fat in the urine, of slight dyspnœa, &c.

4. When prostration and loss of blood have been great, a moderate amount of embolic disturbance of this kind may serve to turn the scales against a patient who would have otherwise recovered.

5. By a proper understanding of this subject certain deaths may be explained which otherwise seem inexplicable.

6. Treatment can only be symptomatic, but may accomplish something.

7. Autopsies should be so conducted as to reveal this condition when present.

### The Oleate of Zinc.

At this particular season, when intertrigo (chafing) is one of the annoyances to which those, particularly in whom there is a redundancy of adipose, are subject, a reference to the therapeutic properties of the zinc oleate is very timely. The subject has been pretty fully covered in an article by Dr. Stelwagen, in a recent number of the *Medical and Surgical Reporter*, of which *Gaillard's Medical Journal* gives the following *resumé* :—

Oleate of zinc is a dry, white, pulverulent, impalpable powder of a soapy touch, resembling powdered soapstone; if pure, it should make a clear solution with oils, lard, &c., over a water-bath. It may be used either as a dusting-powder or as an ointment. An ointment of one or two drachms to the ounce of cosmoline, or any fatty base, is most commonly used. Sometimes the oleate made up in ointment form with oleic acid seems to be more efficacious. A very good way of prescribing it is as follows:—℞. Zinci oleatis; acidi oleici, aa ℨ j; petrolati; cerati simplicis, aa ℨ iij. Ft. ugt. To a great extent this oleate replaces the oxide of zinc, and may be ordered whenever that substance is indicated. Acute vesicular eczema may be successfully treated with the application of black-wash and the subsequent application of an ointment of oleate of zinc; the wash is to be applied with a sponge or soft rag for several minutes, two or three times daily; after each application has dried, a small quantity of the ointment is gently rubbed over. In some instances the disease seems to be more favourably influenced by the oleate employed as a dusting-powder. When such is indicated, the following will prove an eligible formula:—℞. Pulv. zinci oleatis; talci veneti, aa ℨ iij; amyli, ℨ ij. M. This is to be dusted over the parts several times daily. The same plan of treatment is frequently of advantage in all weeping eczemas. In intertrigo, a dusting-powder, such as given above, is very comforting. This oleate makes a harmless toilet-powder, and combined with talc and calamine, as in the formula above, will make an excellent powder for such purpose. ℞. Calaminæ præparatæ, ℨ ij; talci veneti; zinci oleatis, aa ℨ vij; olei rosæ, q. s. M. S.—Toilet-powder. This last may also be employed as a dusting-powder in moist eczema and similar inflammations.—*Therapeutic Gazette*, July, 1884.

### Surgical Follies.

The Tourniquet Folly.—In amputations, except in the rarest circumstances, Esmarch's elastic apparatus should supersede the old-fashioned screw tourniquet of Petit. The latter, while indeed arresting the flow through the arteries, nearly always engorges the parts with venous blood, thus inducing venous hæmorrhage, and causing the seat of operation to be obscured. An even worse folly is that of applying a tourniquet to the femoral or brachial artery to stop bleeding from a crushed leg or forearm while awaiting reaction prior to amputation. Pressure should be made *immediately upon and just above* the crushed tissues, by an elastic or common roller bandage, tightly applied. A tourniquet placed far above the seat of injury on the main trunk interferes with the arterial and venous circulation of the whole limb. Amputation, if by necessity delayed for a few hours, must then be performed through tissues that have become œdematous and liable to gangrene because of the stupidity of the surgeon. Pressure over the crushed structures stops all oozing and free bleeding, and is, probably, less distressing to the patient than the tourniquet applied high up. It can do no harm to the tissues already irretrievably damaged and soon to be removed.

The Aspirator Folly.—The aspirator has, probably, done as much harm by encouraging "shrinking surgery" as it has done good in providing a safe means of puncture for evacuating serous effusions and for establishing diagnosis. Many pus collections are repeatedly aspirated by timid hands and heads when salvation of life demands free incision.

The Drainage-tube Folly.—To leave a drainage-tube or -strand in a wound after the discharge has obtained a widely patulous exit, or, in fact, has nearly ceased, is an egregious folly. Yet the cry for free drainage has, doubtless, led to many commissions of this error, whereby a drainage-tube is allowed to become a seton.—*Philadelphia Polyclinic*, August 15th, 1884.

### A Highly Interesting Record.

In a village, C., near Weimar, where for many years no case of tubercular phthisis had taken place, two years ago several families suddenly discovered one of their members to be suffering from the disease. After a long inquiry, it was discovered by accident that all these families had been buying their spring chickens from one and the same place; viz., from a private hospital in the neighbourhood. A medical student brought the livers of two such chickens to Prof. Johne, in Dresden. The student, whose own sister had become affected with consumption, had lived during his vacation at home with his parents, in C., and he had there at dinner observed the peculiar appearance of the liver of the chickens.

On examination, both organs were found to be full of tubercular bacilli. A thorough investigation was at once instituted, and it was then that the fact came to light that the chickens eaten by the families, members of which had been infected with tuberculosis, had all been bought from the institution mentioned. On further inquiry at the latter place the following facts were elicited:

At about the time when the first case of consumption occurred in the village, an inmate of the hospital, Mrs. R., had died of the disease. Before her death, Mrs. R. used to feed the chickens raised there; she was often seen first to chew the meat before she gave it to the chickens. Further, the spittoons were emptied on a place in the yard where the chickens generally came to pick up any stray corn.

As none of the chickens ever came in contact with any animals in the neighbourhood—the hospital being situated at a considerable distance from the village—as no disease had happened amongst them until the arrival of Mrs. R., when soon after an epidemic seemed to break out amongst them, and many died—there is no doubt that they contracted the disease from Mrs. R., and in return infected those who ate their flesh.

The case is very interesting, first, as it proves how such animals may become affected, then how they may spread the disease; and lastly, that some kind of a disposition must exist in the person infected; for here, of many who had eaten of the diseased flesh, only a few contracted the malady. The whole report teaches us how careful we have to be, and how necessary is the appointment of skilful experts by the State to inspect all food offered for sale.—*Medical and Surgical Reporter*, August 16th, 1884.

### Picrotoxin in the Treatment of Night-Sweats of Phthisis.

Too frequently in the treatment of phthisis the physician's best efforts must be confined to alleviating the symptoms, and, with the prospect of inevitable death before him, hope only to secure tolerance of existence on the part of the patient, and euthanasia. Among the more distressing symptoms or complications which may demand attention are the night-sweats. For the relief of these a great variety of remedies has been from time to time suggested. Drs. Westbrook and Platt, physicians to the Department of Diseases of the Chest in St. Mary's General Hospital, of Brooklyn, New York, contribute to the *New York Medical Journal* the results of their experience with several of the more recent drugs in the many cases in which they were furnished an opportunity for carefully trying them. The drug which they used in the greatest number of cases was picrotoxin. The result of its use in nine cases was to entirely check the sweats in six cases, and to relieve them greatly in two. In one very troublesome case it seemed to have no effect whatever. Their method of administering it was either hypodermically or by the mouth, at intervals of two to ten days, and in doses of from $\frac{1}{60}$ to the $\frac{1}{20}$ of a grain. It was found in some cases that $\frac{1}{20}$ of a grain hypodermically, at night, would control the sweats for ten nights following. These results are corroborative of a favourable report made by Dr. Murrell, of London, who had but one failure in twenty cases, and found the effect to run about ten days. Dr. Murrell, however, accomplished his results with much smaller doses, giving from the $\frac{1}{180}$ to the $\frac{1}{60}$ of a grain only. Atropine was also employed in a number of cases, but although it was tolerably successful as regards the sweats, it was objectionable owing to the annoying dryness of the throat, which followed its use.—*Therapeutic Gazette*, August, 1884.

### The Therapeutic Properties of Buttermilk.

Buttermilk, so generally regarded as a waste product, has latterly been coming somewhat into vogue not only as a nutrient, but as a therapeutic agent; and in an editorial article the Canada *Lancet*, some time ago, highly extolled its virtues. Buttermilk may be roughly described as milk which has lost most of its fat and a small percentage of its casein, and which has become sour by fermentation. Long experience has demonstrated it to be an agent of superior digestibility. It is, indeed, a true milk peptone; that is, milk already partially digested, the coagulation of the coagulable portion being loose and flaky, and not of that firm indigestible nature which is the result of the action of the gastric juice upon sweet cow's milk. It resembles koumiss in its nature, and, with the exception of that article, it is the most grateful, refreshing and digestible of the products of milk. It is a decided laxative to the bowels, a fact which must be borne in mind in the treatment of typhoid fever, and which may be turned to advantage in the treatment of habitual constipation. It is a diuretic, and may be prescribed with advantage in some kidney troubles. Owing to its acidity, combined with its laxative properties, it is believed to exercise a general impression on the liver. It is well adapted to many cases where it is customary to recommend lime-water and milk. It is invaluable in the treatment of diabetes, either exclusively or alternating with skimmed milk. In some cases of gastric ulcer, and cancer of the stomach, it is the only food that can be retained.—*Ibid.*

### Nairne on Statistics.

Do not be deterred by "statistics" from doing what you conceive to be your duty—statistics is a kind of "bogle" set up by the disingenuous, mostly as a signal mark of their own ability, and a dreadful "scare-crow" to others. Distrust them; they may mean anything, they may mean a selection of picked cases, of easy cases, of cases that would have got better under almost anyone's hands, of young, healthy, vigorous patients; they may be an indication certainly of dexterity on the part of the operator, gained by experience, or they may be a freak, a run of successful cases with no special merit to the operator. It is, indeed, high time that this statistical curse were abolished, if it cannot be put on a proper basis, and the miserable quibblings about one man's work, in comparison with another's, brought to a final termination. The justification of any operation is its necessity, and not the numerical recoveries from its performance; but we are all inclined to shirk operation in desperate and unlikely cases when we can, in case we are blamed for hurrying on a fatal termination, or for fear that the patient die under our hands, or immediately after.—*Glasgow Medical Journal*, June, 1884.

### Seiler on the Treatment of Ozœna.

The treatment for atrophic nasal catarrh must be divided into two portions, viz., the removal of the accumulation of secretion and the disinfecting of the nasal cavities to remove the odour, and the stimulation of the mucous membrane, with a view to the regeneration of the serous glands.

This may be accomplished in the following manner:—The nasal cavities are first cleansed with a copious stream of alkaline solution from the nasal douche; and let me tell you here that this is the class of cases in which this much-abused instrument is not only applicable, but extremely useful without being harmful. If after this any plugs remain in the nasal cavities, they must be removed with a pair of forceps, and the washing repeated until all secretion has been removed, as you saw me do in the case before us. The next step is to disinfect the nasal cavities. Various substances have been used for this purpose, such as permanganate of potash, chlorine water, tar water, carbolic acid solution, iodoform, and many others; but they all have either great disadvantages, or else are but partially effectual. Lately I have used a preparation called Listerine, which answers the purpose admirably, without having any of the disadvantages of other disinfectants. It is a combination of the essential oils of thyme, eucalyptus, gaultheria, and other plants, together with a small quantity of benzo-boracic acid, and should be diluted one-half with water when used by the atomizer. The immediate effect is to at once destroy the stench from the nose, and to substitute for it the odour of the oil of thyme, which is rather pleasant than otherwise. I am in the habit of using a spray of this solution before making an examination, or before I cleanse the nasal cavities of such cases, thus mitigating very materially the discomfort to myself arising from the stench. The patient is directed to cleanse his nose with a solution of two drachms of bicarbonate of soda to one quart of tepid water morning and evening, and to throw a spray of the Listerine solution into the nostrils after the cleansing. Treated in this manner, I have seen many cases in which the odour was barely perceptible after the lapse of three or four days, and remained in abeyance throughout the treatment. The oil of thyme and of eucalyptus, besides being disinfectants, have also a stimulating effect upon the mucous membrane, and thus aid in the second portion of the treatment.

The stimulation of the serous glands to a normal action may be brought about by a variety of remedies, such as astringents in various strengths; but in my experience the insufflation into the anterior nasal cavities of finely powdered nitrate of silver, diluted with starch powder, has given the best results. When there is complete absence of the lower turbinated bones, the introduction of a wad of absorbent cotton, which is to remain until washed out, and then be reintroduced by the patient himself, often aids in the stimulation by continually irritating the mucous surface with which it is in contact. Next in effectiveness to the silver powders, I have found a weak solution of ferric alum in the form of a spray thrown into the nasal cavities, and the natural iron water of Cresson springs is peculiarly adapted for these cases.—*Medical and Surgical Reporter*, April 19th, 1884.

### A Plea for More Heroic Surgical Interference in Affections of the Brain.

Dr. R. W. Amidon thus recapitulates an article on this subject in the *Medical News*, June 21st, 1884:

We have trephining, an operation proving fatal in only three per cent. of published cases:

Opening the dura mater, fatal in only 7·6 per cent. of published cases:

We have the brain, an organ tolerant of injury and ready to take on a reparative process:

We are possessed of a knowledge which enables us to tell when certain parts of the brain are diseased; and we also have anatomical data which tell us just where to pierce the envelopes of the brain to reach a certain definite part of its convexity.

We have all the elements of safety and anatomical accuracy: why should cerebral surgery not advance with pulmonary, renal, intestinal, and ovarian surgery?

The substitution of the dental engine and a burr or drill cutting on the side for the ordinary trephine strikes me as advantageous. Its advantage over the trephine is principally that with it you can cut away as much or little bone as you please, and make an opening of any shape you please. It is especially useful in trimming down the edge of over-riding bone, to allow the elevation of depressed fragments.

Let the operation always be done with antiseptic precautions. Try and secure only approximate coäptation of the flaps. Provide the freest possible drainage. Use cold antiseptic dressings, without much compression. Enjoin the strictest quiet in a posture facilitating drainage. Simple diet, and a slightly loose condition of the bowels. On the slightest rise of arterial tension or temperature, give jaborandi or aconite to the production of physiological effects. Quinine and alcohol should, I think, be given only in tonic doses. An anodyne is often indicated, and it is my advice never to use opium, or any of its preparations or derivatives. To ease pain, quiet delirium, or induce sleep, use the hydrate of chloral in small repeated doses (·60–1, every ten or fifteen minutes). Quinine and alcohol in large, and opium in *any* dose, aggravate intracranial inflammation when present, and, I think, may excite it.

These suggestions apply equally well, or with still greater force, to cases in which the dura mater or brain is accidentally or intentionally invaded.

A few words as to what I consider good indications for opening the skull.

An injury of the vault, however slight the marks of external violence, provided there be coma, aphasia, hemiplegia, or hemispasm of the lower part of the face, of the arm, or leg, or all three, constituting hemiepilepsy, whether accompanied or not by chills, fever, headache, and vomiting. General epileptic convulsions do not constitute so good indications for operation. Every case of compound fracture of the skull, whether there be cerebral symptoms or not. Cases in which, after the lapse of months, or years even, unmistakable cerebral symptoms follow an injury to the head. Atrocious and incurable headaches, particularly if localized, aphasia, monoplegias and monospasms, hemiplegic or hemiepileptic seizures or general epileptic attacks, if they have an aura pointing to a localized lesion. In addition to the bone, the dura mater should be opened in all cases in which exploration with a hypodermic needle discloses the products of purulent inflammation or a great deal of fluid blood under it. In all cases in which a serious lesion of the brain is suspected, but cannot be otherwise proven.

In addition to the bone and dura mater, the brain should be explored, delicately with a probe, in all penetrating wounds of its substance, punctured, lacerated, or gunshot. Its mass should be invaded, even when superficially intact, by a fine, blunt, exploring needle, when the presence of foreign bodies or hidden collections of pus are suspected, and, for the extraction or evacuation of such, larger openings should be made with a dull instrument.

Finally, accessible neoplasms of the brain, which have resisted medicinal treatment, and which continue to grow and threaten life, should be removed for the reason that they are generally single, seldom have secondary deposits, are surrounded by an inflammatory zone of demarcation, and *always* kill by pressure.—*Ibid*, July 12th, 1884.

